# *Chlamydia trachomatis* Infection in Pregnancy: The Global Challenge of Preventing Adverse Pregnancy and Infant Outcomes in Sub-Saharan Africa and Asia

**DOI:** 10.1155/2016/9315757

**Published:** 2016-04-06

**Authors:** Kristina Adachi, Karin Nielsen-Saines, Jeffrey D. Klausner

**Affiliations:** ^1^Department of Pediatrics, David Geffen School of Medicine, UCLA, Los Angeles, CA 90024, USA; ^2^Department of Medicine, Division of Infectious Diseases: Global Health, David Geffen School of Medicine, UCLA, Los Angeles, CA 90024, USA; ^3^Department of Epidemiology, Jonathan and Karin Fielding School of Public Health, UCLA, Los Angeles, CA 90024, USA

## Abstract

Screening and treatment of sexually transmitted infections (STIs) in pregnancy represents an overlooked opportunity to improve the health outcomes of women and infants worldwide. Although* Chlamydia trachomatis* is the most common treatable bacterial STI, few countries have routine pregnancy screening and treatment programs. We reviewed the current literature surrounding* Chlamydia trachomatis* in pregnancy, particularly focusing on countries in sub-Saharan Africa and Asia. We discuss possible chlamydial adverse pregnancy and infant health outcomes (miscarriage, stillbirth, ectopic pregnancy, preterm birth, neonatal conjunctivitis, neonatal pneumonia, and other potential effects including HIV perinatal transmission) and review studies of chlamydial screening and treatment in pregnancy, while simultaneously highlighting research from resource-limited countries in sub-Saharan Africa and Asia.

## 1. Introduction

### 1.1. *Chlamydia trachomatis* in Pregnancy: A Global Problem

Over 2 decades following the landmark United Nations' (UN) International Conference on Population and Development in Cairo, which brought unprecedented attention to women's sexual and reproductive health, global statistics continue to reveal a high burden of maternal and infant morbidity and mortality [1, 2]. In 2010, in an attempt to bridge some of these critical gaps, the UN launched the “Global Strategy for Women's and Children's Health” and the “Every Woman Every Child” movement [3]. With renewed commitment to improving the health of the world's most vulnerable women and children, the UN recently released its new “Global Strategy for Women's, Children's, and Adolescents' Health (2016–2030)” this past September 2015 [4].

STIs and pregnancy-related issues are among these historically neglected health problems and continue to be important sources of healthy life years lost for women [1, 5, 6]. In Africa, it is estimated that 92.6 million new cases of the curable STIs (*Chlamydia trachomatis*,* Neisseria gonorrhoeae*,* Treponema pallidum*, and* Trichomonas vaginalis*) occur, whereas 78.5 million and 128.2 million new cases are estimated to be in Southeast Asia and the Western Pacific [7]. As the most common bacterial STI with 105.7 million new cases annually and 100.4 million adults infected at any point in time,* Chlamydia trachomatis* deserves particular attention [7]. In women aged 15–49 years,* C. trachomatis* prevalence rates in these WHO regions are cited as follows: 5.1 million (2.6%) in Africa, 5 million (1.1%) in Southeast Asia, and 20.5 million (4.3%) in the Western Pacific [7].

**Figure 1:**
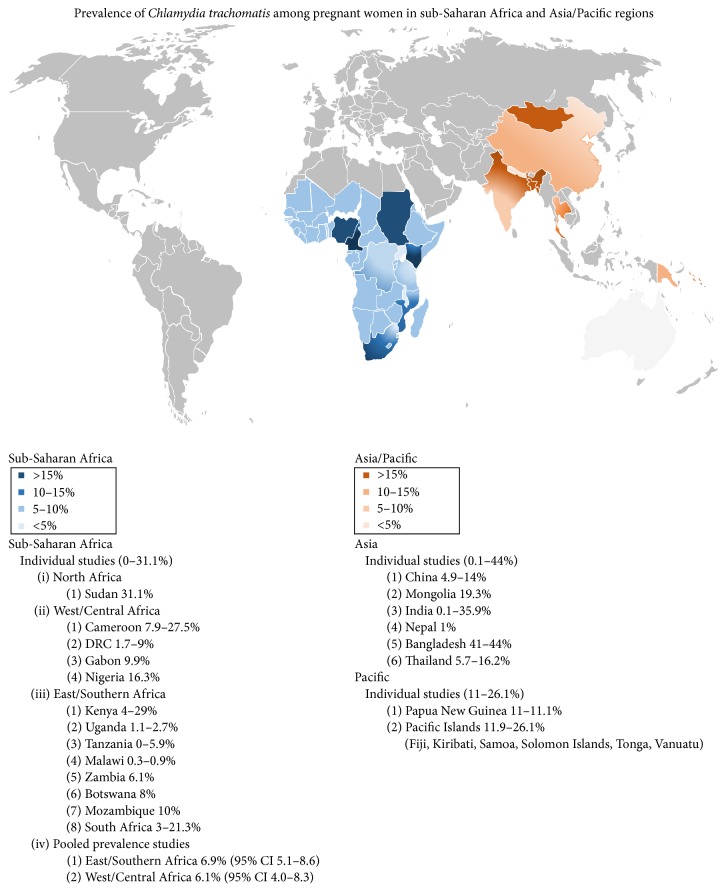
*Chlamydia trachomatis* in pregnant women in sub-Saharan Africa and Asia.

While the global impact of STIs like* C. trachomatis* is felt most acutely by women in poor countries, the consequences may be magnified for pregnant women with potential risks to maternal and infant health. Although limited data exist, worldwide prevalence studies of* C. trachomatis* in pregnant women suggest similar if not higher prevalence rates than in nonpregnant women. Individual studies of pregnant women in sub-Saharan Africa (Sudan, Cameroon, Democratic Republic of Congo (DRC), Gabon, Nigeria, Kenya, Uganda, Tanzania, Malawi, Zambia, Botswana, Mozambique, and South Africa) suggest prevalence rates of 0–31.1%, while pooled prevalence rates are 6.9% (95% CI, 5.1–8.6) in East and Southern Africa and 6.1% (95% CI, 4.0–8.3) in West and Central Africa [8–29]. Other individual studies of pregnant women in China, Mongolia, India, Nepal, Bangladesh, Thailand, Papua New Guinea, and Pacific Islands (Fiji, Kiribati, Samoa, Solomon Islands, Tonga, and Vanuatu) reported rates of 4.9–14%, 19.3%, 0.1–35.9%, 1%, 41–44%, 5.7–16.2%, 11–11.1%, and 11.9–26.1%, respectively [30–51] (Figure 1).

HIV-infected pregnant women may also be at higher risk; one study of HIV-infected pregnant women found* C. trachomatis* prevalence rates of 21.3% for their South African subcohort [24]. Similar findings were seen in a study in Thailand that reported higher rates of chlamydial infection in HIV-infected versus HIV-uninfected pregnant women (16.2% versus 9.1%) [50].

### 1.2. *Chlamydia trachomatis* in Pregnancy: Exploring Adverse Outcomes

As intracellular bacteria with an ability to exist in resting and infectious forms within human epithelial host cells,* Chlamydia trachomatis* presents a unique challenge to eradication [81, 82]. This ability to evade host detection and elimination also contributes to its ability to cause adverse outcomes among women. While it is often an asymptomatic infection in women,* C. trachomatis* is also an important cause of cervicitis, urethritis, and pelvic inflammatory disease (PID), which is an ascending infection of the uterus, fallopian tubes, or neighboring pelvic structures that can vary in presentation as asymptomatic endometritis, salpingitis, tuboovarian abscess, pelvic peritonitis, perihepatitis, or periappendicitis [81].

In addition, infection with* C. trachomatis* may pose special risks for pregnant women.* Chlamydia trachomatis* and other reproductive tract infections have long been suspected as risk factors for adverse pregnancy outcomes [83]. Further support comes from the fact that other* Chlamydia* species apart from* C. trachomatis* (*C. pneumoniae* and* C. abortus*) as well as* Chlamydia*-like emerging organisms (*Waddlia* and* Parachlamydia*) have also been linked with adverse pregnancy outcomes, especially for miscarriage, stillbirth, and preterm birth [84–86].

Both epidemiologic and experimental studies have suggested that chlamydial infection during pregnancy poses a risk for adverse outcomes such as miscarriage (pregnancy that ends spontaneously before the fetus has reached a viable gestational age of 24 weeks), stillbirth (fetal death at 28 or more weeks of gestation), and preterm birth (birth before 37 weeks' gestation) by either direct fetal infection, placental damage, or severe maternal illness. (Note that the gestational age cutoffs for stillbirths vary; in the USA, 20 weeks' gestational age is used; however, the International Classification of Diseases and Related Health Problems, 10th revision, uses the cutoff of 22 weeks' gestational age or birth weight of 500 g; in contrast, the WHO uses a cutoff of 28 weeks or 1000 g because, in many low- and middle-income countries, many infants will not survive if born before 28 weeks' gestation [85–88]). The mechanism by which chlamydial infection may lead to adverse outcomes in pregnancy is not well understood. It is thought that* C. trachomatis* may infect the fetus, triggering a harmful inflammatory response with cytokine release leading to miscarriage, premature rupture of membranes, or preterm labor or possibly causing a maternal inflammatory response that induces embryonic rejection due to homology of the chlamydial and human 60 kDa heat shock proteins [85, 86, 89, 90]. It has also been hypothesized that these inflammatory responses to chlamydial heat shock protein (CHSP-60) may also be responsible for tubal damage that may lead to tubal infertility and ectopic pregnancy [91–93].

## 2. Adverse Pregnancy Outcomes

### 2.1. Miscarriage, Stillbirth, and Ectopic Pregnancy

Some have suggested that infections in general may account for up to 10–66% of late miscarriages and may be a more prominent risk factor for miscarriage in pregnant women in low- and middle-income countries [94, 95]. Genital tract infections such as* C. trachomatis* have been cited as a potential trigger of miscarriages [86, 94–97]. However, the strength of evidence linking STIs with miscarriage is most notable for syphilis and HIV [98–100].

Studies investigating the role that* C. trachomatis* may play in miscarriages have had mixed results, including a small meta-analysis of 4 studies that failed to support an association [32, 101–104]. Some studies have documented associations between miscarriage and prior chlamydial infection as demonstrated by anti-chlamydial IgG and IgA antibodies and detection of chlamydial DNA/antigen from products of conception and placentas of miscarriages [52, 85, 105, 106]; one study found that women with positive chlamydial serology were more likely to have miscarriages than controls (aOR 2.3, 95% CI 1.1–4.9), and* C. trachomatis* DNA was more common in products of conception and the placenta in women with miscarriage than in controls (4% versus 0.7%, and *p* = 0.026) [105]. Scarce data exist from sub-Saharan Africa and Asia regarding the role of* C. trachomatis* in miscarriage, apart from one study from China and another from India [32, 52] (Table 1).

While more global data on stillbirth exist in comparison to miscarriage, global stillbirth estimates remain uncertain due to the lack of accurate data. Since almost half of the world's 130 million births occur at home, global stillbirth estimates were nearly nonexistent before 2006 [87]. It has been estimated that almost 99% of stillbirths occur in low- and middle-income countries [87]. In regions such as South Asia and sub-Saharan Africa, rates may be as high as 32–34 per 1,000 births, which contrasts with that of 3.1 per 1,000 births in high-income countries [107–109].

Maternal infections are thought to be important causes of stillbirth, accounting for half of stillbirths in low- and middle-income and 10–25% in high-income countries [86, 110, 111]. Although published information on STIs other than syphilis is scarce, some studies have suggested that chlamydial infection may also play a role in stillbirths [100]. One such study found* C. trachomatis* antibodies in 33.3% of mothers with stillbirths in comparison to 10.4% of mothers with live births (*p* < 0.0005) (note that stillbirth was defined as greater than 21 weeks' gestational age; based on some definitions such as the WHO definition of stillbirth, this would be classified as miscarriage as opposed to stillbirth) [112]; similar findings were reported in another large cohort of pregnant women, where chlamydial infection was a predictor for perinatal death [106, 113, 114]. Others have recovered* C. trachomatis* from the amniotic fluid and fetal lung and liver tissue from women suffering pregnancy loss with intact membranes [86, 106, 115]. Another retrospective study found that women with chlamydial infection prior to birth were at higher risk for stillbirth (aOR 1.40, 95% CI 1.00–1.96) [116]. Only one individual study of chlamydial infection and stillbirth from Asia or sub-Saharan Africa was identified and reported higher rates of intrauterine death among Indian women with positive chlamydial serology (4.7% versus 0%) [37] (Table 1).

Ectopic pregnancy, which occurs when the blastocyst implants outside of the uterus endometrial cavity (in either the fallopian tubes, ovaries, or abdomen), may complicate 1–3% of pregnancies [92, 117–119]. Ectopic pregnancy can be a life-threatening condition and remains an important global cause of maternal morbidity and mortality due to associated complications such as tubal rupture and hemorrhage [117, 118, 120–124]. Some studies from sub-Saharan Africa (Cameroon, Ghana, and Mozambique) have reported that ectopic pregnancy may account for 3.6–12.5% of cases of maternal deaths [121–123].

Prior ascending genital tract infections leading to pelvic inflammatory disease have been considered risk factors for tubal damage that can lead to ectopic pregnancy and tubal infertility; some have suggested that genital infections may pose a threefold to fourfold increased risk of developing ectopic pregnancy [118, 119]. The proposed association between genital tract infections, particularly* Chlamydia trachomatis*, and the development of ectopic pregnancy primarily results from epidemiological studies reporting recovery of chlamydial antibodies (including antibodies specific to* C. trachomatis* and chlamydial heat shock protein (CHSP-60)), antigenic material, and histologic evidence more frequently from women with ectopic pregnancies versus controls [93, 106, 125–131].

In addition, the results from several studies from countries in sub-Saharan Africa (Gabon, Senegal, and Zimbabwe) and Asia (Myanmar and Vietnam) also suggest that* C. trachomatis* may be a risk factor for ectopic pregnancy [54–58] (Table 1).

### 2.2. Preterm Labor, Preterm Birth, and Low Birth Weight

Globally, preterm birth has been identified as the single most important cause of perinatal morbidity and mortality, accounting for 27% of the nearly four million reported annual neonatal deaths; it is a risk factor for chronic lung disease, infections, and neurologic disabilities including intracranial hemorrhage, cerebral white matter damage, and cerebral palsy [86, 107, 111, 132, 133]. Around the world, preterm birth has also recently become the leading contributor to mortality for all children under five, not just neonates [134–136].

Worldwide studies have estimated that 14.9 million infants are born premature [107, 135, 137]. Estimates of preterm birth rates range from 5% in Europe to 18% in African nations with an average of 7.5%, 8.8%, and 12.5% in more, less, and least developed regions [107, 135, 137]. Yet, it is in sub-Saharan Africa and South Asia, where over 60% of the preterm infants are born; India, China, Nigeria, Pakistan, and Indonesia have the highest numbers of preterm births in the world [135].

While the vast majority of preterm births occur secondary to spontaneous preterm labor, preterm birth is often the end product of numerous causal factors [137]. Some have suspected that genital tract infections may be a risk factor for preterm delivery. Symptomatic and chronic intrauterine infection with organisms like* C. trachomatis* may be important contributing factors with some suggesting that genital tract infections may contribute to as many as 40% of preterm birth cases [86, 138].

While some studies have shown no significant association between* C. trachomatis* and preterm birth [21, 38, 41, 43, 139–144], the majority of studies reviewed suggest that chlamydial infections increase the risk for preterm delivery and/or low birth weight [9, 22, 30, 37, 59, 60, 84, 112–114, 116, 132, 145–164]. Those findings are well-summarized in a 12-study meta-analysis reporting that chlamydial infection during pregnancy was associated with an increased risk of preterm labor (RR 1.35, 95% CI 1.11–1.63), low birth weight (RR 1.52, 95% CI 1.24–1.87), and perinatal mortality (RR 1.84, 95% CI 1.15–2.94) [101].

Prospective studies have found that placental inflammation (OR 2.1, 95% CI 1.2–3.5) and chlamydial DNA were more frequently isolated from placentas of women who delivered at or before 32 weeks [132]. Similarly, other studies have suggested that maternal chlamydial infection may increase the risk of preterm delivery (RR 1.46, 95% CI 1.08–1.99) and premature rupture of membranes (RR 1.50, 95% CI 1.03–2.17) [162], and a large population-based prospective study of 4,055 pregnant women reported that chlamydial infection was associated with more than a fourfold increased risk of early preterm delivery (OR 4.35, 95% CI 1.3–15.2) [164]. Another frequently cited case-control study, which analyzed urine specimens for* C. trachomatis* from 190 women with preterm birth, observed that chlamydial-infected women at 24 weeks of pregnancy were 2 times more likely to have spontaneous preterm birth <37 weeks (OR 2.2, 95% CI 1.03–4.78) and three times more likely to have an early spontaneous preterm birth <35 weeks of gestation (OR 3.2, 95% CI 1.08–9.57) [163]. However, support of those findings was not observed in another secondary analysis by the same authors [144].

Despite the substantial burden of preterm birth estimated in sub-Saharan Africa and Asia, few published studies of* C. trachomatis* and related outcomes of preterm labor and/or low birth weight from countries in these regions exist. Among the studies that could be identified, the majority seem to support a role for* C. trachomatis* in preterm birth and similar outcomes. Two of the twelve studies included in the meta-analysis discussed above were from Asia [30, 37]. One was a study of 300 pregnant women in China, which found higher rates of premature rupture of membranes in women with chlamydial infection compared to those without (30.3% versus 13.5%, *p* < 0.05) [30]. The other was a small study of 78 Indian women, which observed that women with positive* C. trachomatis* serology had higher rates of preterm labor (9.7% versus 0%) and low birth weight infants (28.7% versus 2.6%) compared to those with negative serology [37]. Several other studies from South Africa [22, 59] support those findings as well as a study from Cameroon, where pregnant women with chlamydial infection were almost three times more likely to have preterm labor (OR 2.8, 95% CI 1.1–6.97) [9]. In contrast, a few studies, including ones from India, Nepal, and South Africa, did not find significant associations, possibly due to issues with sample size and/or low prevalence of* C. trachomatis* [21, 38, 41, 43] (Table 1).

A recent secondary analysis of 1373 HIV-infected pregnant women (approximately 30% of the cohort included South African women with a high prevalence of both* C. trachomatis* and* Neisseria gonorrhoeae*) also demonstrated significant differences in rates of infant low birth weight (42.9% versus 16.9%, *p* = 0.001) and preterm birth (28.6% versus 10.2%, *p* = 0.008) for women with and without these STIs [60, 165] (Table 2).

### 2.3. Prevention of Adverse Pregnancy Outcomes

Given that* Chlamydia trachomatis* and other STIs are curable infections, many pregnancy and neonatal complications could potentially be prevented with antenatal screening programs that accurately identify and treat infected women [153, 154, 166].

However, only a small number of studies have attempted to evaluate the potential benefits of chlamydial antenatal screening and treatment to prevent adverse pregnancy outcomes such as low birth weight, preterm delivery, preterm labor, or premature rupture of membranes [26, 39, 144, 152–154, 166, 167]. These studies varied with respect to study design, method of testing, collected specimen type, gestational age at testing, number of other STIs evaluated, and antibiotic used for treatment. All of those studies except one [144] provided some level of support that screening and treatment of chlamydial infection in pregnancy could improve rates of adverse pregnancy outcomes [26, 39, 152–154, 166, 167]. Only two of the studies took place outside of the USA, including one in Uganda and one in India [26, 39] (Table 3).

Four of those studies presented the strongest evidence suggesting that chlamydial treatment may lead to improved pregnancy outcomes [152–154, 166]. Some of the studies found significant reductions in preterm birth [13.9% to 2.9%, *p* = 0.00002, OR 0.16, 95% CI 0.06–0.47] [153]; premature rupture of membranes [5.2% to 2.9% (OR 0.56, 95% CI 0.37–0.85) [154], 20.3% to 7.4% (OR 0.31, 95% CI 0.14–0.69) [153], and 50% to 0% (RR 0.4, 95% CI 0.2–0.8)] [152]; and/or low birth weight infants [17% to 8%, *p* = 0.04 [166], and 19.6% to 11%, *p* < 0.0001] [154] when comparing women treated for chlamydial infection versus untreated or persistently infected women. Yet, those studies also had limitations including the antibiotic regimen used, failure to directly treat partners, unknown usage of other antibiotics, and failure to see significant findings in one of the study's preliminary analyses.

## 3. Adverse Infant Outcomes

Studies dating back to the 1970s demonstrated that* Chlamydia trachomatis* could be vertically transmitted at the time of delivery from mothers to infants [168]. Earlier studies estimated that approximately 50–70% of infants born to mothers with untreated genital chlamydial infection will become infected with 30–50% developing conjunctivitis and 10–20% developing pneumonia [169–171]. In studies from sub-Saharan Africa (2 from Kenya) and Asia (4 from China, 1 from Thailand), most have found similarly high rates of* C. trachomatis* vertical transmission [13, 30, 33–35, 49, 61] (Table 2).

The 1980s implementation of antenatal screening and treatment for chlamydial infection in the USA significantly lowered the incidence of both neonatal chlamydial pneumonia and conjunctivitis, which was previously the most common cause of neonatal conjunctivitis there [169]. Due to the lack of similar initiatives in other countries, chlamydial conjunctivitis and pneumonia continue to be prevalent worldwide [169, 172]. Compared to the sparse data on adverse pregnancy outcomes and* C. trachomatis* from sub-Saharan Africa and Asia, more published information documenting infant chlamydial infection from countries in these regions exists (Table 2).

### 3.1. Conjunctivitis

Classically, chlamydial conjunctivitis develops 5–14 days after birth with symptoms ranging from mild conjunctival injection with discharge to severe mucopurulent conjunctivitis with chemosis and pseudomembrane formation [173, 174]. Although vision loss is rare, consequences of untreated infection include persistent conjunctivitis, pannus (neovascularization of the cornea), and scarring [173]. Differing from gonococcal conjunctivitis, chlamydial conjunctivitis cannot be effectively prevented using antibiotic or silver nitrate ocular prophylaxis [169, 174, 175].

Existing studies from sub-Saharan Africa and Asia also suggest that* C. trachomatis* remains a frequent cause of neonatal conjunctivitis. Studies of infants from sub-Saharan Africa (Cameroon, Gabon, Gambia, and Kenya) have demonstrated that* C. trachomatis* remains a common etiology of ophthalmia neonatorum and may account for up to 33% of cases [13, 68–72]. Studies from China have estimated that chlamydial conjunctivitis occurs in 4 per 1,000 live births [64, 169]. Apart from one study from Singapore [66], other studies from Asia (China, Cambodia, and Thailand) also report frequently isolating* C. trachomatis* in 12.2%–60% of infants with conjunctivitis or ophthalmia neonatorum [62–65, 67] (Table 2).

### 3.2. Pneumonia

Being often underdiagnosed, chlamydial pneumonia tends to be a subacute, afebrile infection, typically occurring in infants between 1 and 3 months of age [174]. In younger infants, especially in the premature, chlamydial pneumonia can be more severe, associated with apnea, and may require hospitalization in 25% [173, 176]. Although associated mortality is supposedly rare, untreated pneumonia can persist for several weeks and may lead to poor feeding and diminished weight gain; some have suggested that infection may lead to asthma and chronic lung disease later in life [173, 177, 178].

As in studies from other countries, existing studies of infants from sub-Saharan Africa have suggested that* C. trachomatis* may be a frequent but underrecognized pathogen in lower respiratory tract infections including pneumonia [169, 179]. Most studies evaluating infants from Ethiopia, Kenya, and South Africa found that* C. trachomatis* was a frequent isolate (6–51%) from infants with lower respiratory tract infections including pneumonia [77–80]. The study from Ethiopia found that* C. trachomatis* was the 2nd most common infectious etiology (15.8%) after* Respiratory Syncytial Virus* (RSV) (28%) in infants less than 3 months presenting with pneumonia; similar findings were also reported in a study of infants in Netherlands (7%) and a study of infants in Thailand (18.5%) [74, 77, 169, 179]. Excluding the findings of a small study of Malaysian children with pneumonia, other studies from Asia (Thailand) also emphasized the importance of chlamydial infection in young children with pneumonia as well as a possible coinfection pathogen for those with RSV bronchiolitis [73, 75, 76] (Table 2).

### 3.3. Other Adverse Infant Outcomes

Beyond the well-documented risks of neonatal chlamydial infection (conjunctivitis and/or pneumonia) associated with maternal chlamydial infection in pregnancy, some studies suggest other consequences of untreated STIs in pregnancy. A few studies have observed increased rates of neonatal and infant death with STIs such as* C. trachomatis* in pregnancy [60, 101, 146, 165, 180, 181] (Table 2).

*Possible Risk Factor for HIV Mother-to-Child Transmission (MTCT)*. Concern also exists that STIs may increase the risk of HIV mother-to-child transmission (MTCT), for early studies have suggested that genital infections like* C. trachomatis* may lead to increased cervicovaginal shedding of HIV and chorioamnionitis [182–186]. However, few published studies have explored the effect that STIs such as* C. trachomatis* in pregnancy may have on HIV MTCT. In one of the HIV Prevention Trials Network (HPTN) 040 substudies of 1373 HIV-infected pregnant women, the rates of HIV MTCT among women infected with* C. trachomatis* (10.7%) were significantly higher compared to those uninfected (8.1%); further analysis also suggested a possible association of chlamydial infection and increased HIV MTCT (OR 1.47, 95% CI 0.9–2.3; *p* = 0.09) [24] (Table 2).

Yet, those findings of increased risk of HIV MTCT were not observed in a smaller study from Thailand of 222 HIV-infected pregnant women in spite of a high prevalence of chlamydia (16.2%) [50]. The findings of the HPTN 040 substudy were also not supported by findings of earlier randomized trials of empiric STI treatment during pregnancy including the well-known Rakai study in Uganda and the HPTN 024 study that took place in Zambia, Malawi, and Tanzania; both studies failed to demonstrate that empiric antibiotics effective against* C. trachomatis* had any impact on reducing the rates of HIV MTCT [26, 184]. Both studies had low rates of* C. trachomatis* in their study populations (1.1–2.7% and 2.5%), which may have contributed to the lack of findings [15, 26, 183, 184].

### 3.4. Prevention of Infant Chlamydial Infection

Screening and treatment of chlamydia in pregnancy has been considered by some as the only effective means of preventing chlamydial pneumonia, conjunctivitis, or colonization in infants [169]. Yet, in the existing literature, only a handful of primary studies have provided information regarding the effect of screening and treatment on prevention of neonatal chlamydial infection, and none of these studies took place in sub-Saharan Africa or Asia [141, 168, 170, 187–190]. Almost all of the studies used erythromycin as the primary therapeutic intervention or as part of the interventions evaluated, and nearly all used chlamydial cervical culture to evaluate for infection in women. Wide variability was noted in study design, cohort size, chlamydia prevalence, time of testing, time of therapeutic intervention, and methods used to evaluate for infant chlamydial infection.

All of these studies, except one small study of 21 women, found significant differences in rates of infant chlamydia for women treated for chlamydial infection during pregnancy [190]. Two observational studies from the mid-1980s in the USA provided the strongest evidence that antenatal chlamydial treatment with erythromycin may decrease chlamydial infection in infants [168, 170]. These studies observed significantly lower rates of infant chlamydial infection for those born to women receiving treatment with erythromycin as opposed to no treatment for chlamydia; infant chlamydial infection decreased from 50% (12/24) to 7% (4/59) (*p* < 0.001) in one and from 23.8% (5/21) to 0% (0/16) (*p* < 0.04) with treatment in the other. Infants of untreated women were also more likely to have symptomatic infection with conjunctivitis and pneumonia. These studies were not without limitations, which included considerable numbers of women and infants lost to follow-up and the use of a nonstandardized erythromycin treatment regimen in one of the studies.

## 4. Preventing Chlamydial Adverse Pregnancy and Infant Outcomes: Vaccine Development and Antenatal Screening and Treatment

### 4.1. Global and Historical Challenges

Worldwide prevention of adverse pregnancy and neonatal outcomes from* Chlamydia trachomatis* has been impeded by two primary factors: lack of an effective human vaccine and lack of progressive, targeted screening/treatment recommendations for pregnant women. The development of a safe and effective vaccine would likely provide the best hope of reducing the global burden of disease from* C. trachomatis*, especially its associated maternal and infant morbidity that can even result from asymptomatic infections. Yet, historically, the development of a* C. trachomatis* vaccine has been riddled with challenges, which were noted even with the early human vaccine trials in the 1960s [191]. These early immunization trials of whole organism preparations had issues with waning immunity and raised concerns about the risks of immunopathology and the potential for reversion back to wild-type strains [192]. Vaccine efforts since then have focused on other targets such as major outer membrane proteins (MOMP) and chlamydial outer membrane complex (COMC) proteins while hunting for alternative options [192–194]. The use of new candidate antigen targets such as polymorphic membrane proteins (PMPs), incorporation of additional promising vaccine targets such as dendritic cells, and the discovery of novel, less toxic adjuvants may provide greater opportunities to develop a successful human vaccine in the upcoming years [192, 194–196].

Historically, considerable obstacles have also thwarted efforts to improve global chlamydial screening and treatment practices for pregnant women. In spite of improved detection of chlamydial infections with molecular-based nucleic acid testing, more patient friendly specimen collection methods, and simple, highly effective, one-dose oral treatment regimens, few countries around the world have made* Chlamydia trachomatis* screening and treatment a priority for pregnant women [197–201]. While some such as the USA have recommended universal* C. trachomatis* screening and treatment for all pregnant women or those at high risk since the 1980s, this is not standard practice globally [197, 202, 203].

In low- and middle-income countries including those in sub-Saharan Africa and Asia, routine* C. trachomatis* screening during pregnancy has been previously hindered by limited awareness and lack of access to diagnostic methods [2, 7]. Traditionally, in resource-limited settings, the diagnosis of* C. trachomatis* and other STIs has focused on a “syndromic approach,” which still remains endorsed by the WHO. This “syndromic approach” is notorious for its low sensitivity (30–80%), performing particularly poor for* C. trachomatis* detection, which is typically asymptomatic [5, 14, 29, 204–206]. Those findings were emphasized in a recent South African study of 1480 women that found that more than 50% of* C. trachomatis* and other STIs were asymptomatic [23].

### 4.2. Antenatal Chlamydial Screening

Early successes of integrating antenatal screening and treatment of syphilis with existing HIV prevention of mother-to-child transmission (PMTCT) programs have shown the potential to expand such programs to include screening and treatment of other STIs such as* C. trachomatis*. Studies, particularly from China, have highlighted the ability for countries to lead rapid antenatal screening scale-up interventions. Some Chinese provinces have demonstrated near-universal screening rates for HIV, hepatitis B, and syphilis with significant reductions in congenital syphilis cases as well as adverse pregnancy outcomes including miscarriage, premature delivery, and stillbirth [207–209].

*Need for Point-of-Care Testing*. Much of the tolerance for the continued reliance on the “syndromic approach” to address* C. trachomatis* and other STIs in resource-limited countries has been a result of the cost and infrastructure required for the implementation of current testing methods used in resource rich countries.

Since* C. trachomatis* is an intracellular organism, older methods of identification relied on culture and enzyme immunoassay, which are labor intensive and demand considerable training, laboratory resources, and costs [210]. Currently the recommended tests for the detection of* C. trachomatis* are nucleic acid amplification tests (NAATs) [211, 212]. NAATs for* C. trachomatis* have high specificity (98–100%) and high sensitivity (95%) and can be used on noninvasive specimens such as urine and self-collected vaginal swabs [210].

With the introduction of NAATs for the detection of HIV and* Mycobacterium tuberculosis* in most countries around the world, the use of NAATs for* C. trachomatis* detection is highly feasible, even though they are not considered “point-of-care” (POC) tests [213]. Among the newer nucleic acid testing platforms recently developed is the Cepheid GeneXpert CT/NG assay, which has been considered a “near-patient” testing method but not truly rapid (<20 minutes) or of low cost [213]. It uses real-time PCR with a cartridge assay and features easy specimen loading with results in 90 minutes along with a record of excellent sensitivity and specificity (97–98.7% and 99.4–99.9%).

The currently available POC tests for* C. trachomatis* are mainly poorly sensitive optical immunoassays (OIA) such as* Inverness* (previously* BioStar*),* Clearview Chlamydia*,* QuickVue*,* Chlamydia Rapid Test*, and* OneStep* and magnetic immunochromatographic tests that utilize rapid platforms based on antigen/antibody interactions [213]. One study of women tested in clinics in the Philippines, which compared a* C. trachomatis* POC test with a NAAT, found extremely poor sensitivities (12.5–19.4%); poor sensitivity of* C. trachomatis* POC testing was also found in a study in six urban cities in China (32.8–49.7%) [214, 215].

## 5. Conclusion

Untreated STIs in pregnancy, particularly* Chlamydia trachomatis*, continue to negatively impact the health of women and infants worldwide given the lack of a* C. trachomatis* vaccine and lack of adequate STI screening and treatment policies in most countries. There exists a great need to develop an effective chlamydial vaccine and to also incorporate antenatal screening for STIs such as* C. trachomatis* and* Neisseria gonorrhoeae* and possibly others such as* Group B Streptococcus* (GBS) and hepatitis B with existing networks currently conducting successful HIV and syphilis antenatal screening [216]. Studies from Zambia and China have suggested that an integrated approach to antenatal care that builds on existing HIV prevention of mother-to-child transmission (PMTCT) platforms has multiple benefits beyond just reduction in HIV vertical transmission; it capitalizes on the ability to address other important infections in pregnancy and increases antenatal care attendance [217, 218].

While few high-quality studies have investigated the benefit of antenatal chlamydial screening and treatment, the collective evidence appears to support the possibility of benefits for such an intervention. Large-scale, randomized clinical trials to investigate the true impact and cost-effectiveness of screening and treatment initiatives to improve pregnancy and infant outcomes are urgently needed. Hopefully, the continued evolution of better evidence and increasing availability of* C. trachomatis* detection assays will eventually persuade policy makers to address this neglected problem of STI screening and treatment in pregnancy [197, 219–221].

We can no longer afford to fail to invest in the sexual and reproductive health of women. The problem of STIs in pregnancy is a health issue affecting women, children, and adolescents. Building the evidence base for screening and treatment of STIs like* Chlamydia trachomatis* in pregnancy should be made a global priority because the health of “every woman, every child” matters [3, 4].

## Figures and Tables

**Table 1:** Studies from sub-Saharan Africa and Asia on *Chlamydia trachomatis* and adverse pregnancy outcomes.

CT and adverse pregnancy outcomes (total studies = 17)				
Study	Region	Country	Support association	Findings
*CT and miscarriage* (studies = 2)				

Li et al., 2010 [32]	Asia	China	No	Compared 74 women with spontaneous abortion during the 1st or 2nd trimester with 62 women with induced abortion. No difference in CT rates in spontaneous versus induced abortion groups (8.11% versus 8.06%, *p* = 0.91)
				
Rastogi et al., 2000 [52]	Asia	India	Yes	Compared 77 women with spontaneous abortion at 6–24 weeks' gestation with 25 women at 6–16 weeks' gestation with induced abortion. CT was recovered more frequently among women with spontaneous compared to induced abortion (15.6% versus 4%)

*CT and stillbirth* (studies = 1)				

^*∗*^Jain et al., 1991 [37] (see below)				

*CT and ectopic pregnancy* (studies = 6)				

Zhu et al., 2014 [53]	Asia	Shanghai, China	No	Compared 71 women with ovarian pregnancy, 145 women with tubal pregnancy, and 146 women with intrauterine pregnancy (controls). Differences were noted in rates of serological evidence of CT between groups of women with ovarian pregnancy (14.9%), tubal pregnancy (34%), and intrauterine pregnancy (9.9%), *p* < 0.01, but a significant association was not seen when comparing ovarian to intrauterine pregnancy
				
Khin Nwe et al., 2011 [54]	Asia	Myanmar	Yes	Evaluated 113 women with ectopic pregnancy versus 226 controls with spontaneous miscarriage and tested cervical and tubal samples for CT (also evaluated for gonorrhea and syphilis). CT DNA from cervical swabs was more frequently recovered from ectopic pregnancy cases versus controls (8% versus 2.2%, *p* = 0.01); CT recovered from 15% of tubal samples from women with ectopic pregnancy
				
Hornung et al., 2015 [55]	Asia	Ho Chi Minh City, Vietnam	Yes	Case-control study of 343 women evaluated for CT and *Waddlia chondrophila*. CT IgG was associated with ectopic pregnancy (aOR 5.4, 95% CI 2.6–11.3), but no CT DNA was recovered from tubal lesions
				
Ville et al., 1991 [56]	Africa	Franceville, Gabon	Yes	84% of women with ectopic pregnancy versus 53% of controls (5–12 weeks' gestation) and 39% of controls (32–41 weeks' gestation) had positive CT serology, *p* < 0.0001. CT recovered from tube cultures in 71% of ectopic pregnancies; positive CT serology associated with pelvic adhesions
				
Cisse et al., 2002 [57]	Africa	Dakar, Senegal	Yes	Retrospective study of 337 women with salpingectomy for ectopic pregnancy and recovered CT in 23.4% of cases
				
De Muylder 1991 [58]	Africa	Gweru, Zimbabwe	Yes	Compared CT serology in 104 women with ectopic pregnancies versus 90 controls (those with full-term pregnancies). Significantly higher rates of CT antibodies (titer ≥ 1 : 16) seen in women with ectopic pregnancy and abnormal tubes (69%) compared with ectopic pregnancy and normal tubes (22%) versus intrauterine pregnancy controls (38%), *p* < 0.01. Similar findings seen for those with higher CT titer antibodies (≥1 : 64) for women with ectopic pregnancy and abnormal tubes (26%), ectopic pregnancy and normal tubes (0%) versus controls (4%), *p* < 0.01

*CT and preterm rupture of membranes, preterm labor, and/or preterm birth* (studies = 9)				

Yu et al., 2009 [30]	Asia	Chongqing, China	Yes	Tested 300 pregnant women for CT and found higher rates of PROM among CT-infected compared to CT-uninfected women (30.3% versus 13.5%, *p* < 0.05)
				
Jain et al., 1991 [37]	Asia	Lucknow, India	Yes	Evaluated 78 pregnant women in the 3rd trimester for CT and found higher rates of premature labor (9.7% versus 0%), low birth weight infants (28.7% versus 2.6%), *p* < 0.05 and intrauterine death (4.7% versus 0%) among CT-infected versus uninfected women
				
Paul et al., 1999 [38]	Asia	New Delhi, India	No	Two cohorts evaluated for CT among pregnant women: (1) 94 women at 26–30 weeks' gestation evaluated for CT, (2) 172 women evaluated at labor for CT. Mean (SD) birth weight [2869 (611) g versus 2814 (496) g], gestation [38.5 (2.6) weeks versus 38.3 (2.0) weeks], low birth weight [18.7% versus 20.7%], and prematurity rates (9.4% versus 10.7%) were similar among infants of CT-infected and uninfected women. Purulent conjunctivitis was more frequent among infants born to CT-infected versus uninfected women (12.5% versus 2.8%, *p* = 0.04)
				
Alexander et al., 1993 [41]	Asia	Vellore, India	No	Evaluated 273 pregnant women at 26–36 weeks' gestation for CT (3.3%) and pregnancy outcomes were followed. Rates of preterm labor (14.3% versus 3.5%), PROM (28.6% versus 17.5%), and low birth weight infants (14.3% versus 11.5%) were higher among CT-infected women compared to uninfected women but were not significantly different
				
Christian et al., 2005 [43]	Asia	Sarlahi, Nepal	No	Evaluated 1177 postpartum women in a secondary analysis of a micronutrient supplement trial. No difference was found in preterm birth (30% versus 20.7%; OR 1.6, 95% CI 0.4–6.4) and low birth weight (40% versus 38.4%; OR 1.1, 95% CI 0.2–4.6) among CT-infected and uninfected women. Eye discharge was only associated with gonorrhea not CT
				
Ngassa and Egbe, 1994 [9]	Africa	Yaounde, Cameroon	Yes	Evaluated 126 pregnant women between 28 and 34 weeks' gestation and found that CT-infected women were more likely than CT-uninfected women to have preterm labor (OR 2.8, 95% CI 1.1–6.97)
				
van Rensburg and Odendaal 1992 [22]	Africa	Cape Town, South Africa	Yes	Evaluated 206 pregnant women for CT and other infections and found that CT-infected women had higher rates of premature deliveries of 47% (8/17) versus 17% (28/163) in CT-uninfected women, *p* < 0.003
				
Donders et al., 1991 [59]	Africa	Pretoria, South Africa	Yes	Evaluated infant outcomes in 11 women with CT cervicitis and 13 women without CT and found lower birth weights (2446 g versus 3017 g, *p* < 0.01) among CT-infected women but no significant differences in mean gestational age (36.8 versus 38.5 weeks, *p* > 0.05) or rates of premature delivery (45.5% versus 23.1%). Also evaluated other infant outcomes: severe neonatal infection (27.3% versus 0%). No CT was recovered from infant conjunctival swabs from either group
				
Donders et al., 1993 [21]	Africa	Pretoria, South Africa	No	Evaluated 256 women at the first antenatal visit as part of a larger cervicitis pregnancy study, and CT was not associated with low birth weight (2803 g versus 2919 g) or premature delivery (27% versus 16%, RR 2, 95% CI 0.6–6.1)

Refer to Table 2 for further details on study by Adachi et al. [60], regarding CT, preterm delivery, and low birth weight.				

CT = *Chlamydia trachomatis.* PROM = premature rupture of membranes, aOR= adjusted odds ratio.

**Table 2:** Studies from sub-Saharan Africa and Asia on *Chlamydia trachomatis *on adverse infant outcomes.

CT and adverse infant outcomes (total studies = 29)				
Study	Region	Country	Support association	Findings
*CT and vertical transmission* (studies = 7)				

Zhang et al., 1994 [35]	Asia	China	Yes	130 pregnant women were evaluated for CT and infants were followed up for 2–6 months with 8.6% of infants noted to have palpebral infection
				
Shen et al., 1995 [33], and Wu et al., 1999 [34]	Asia	Chongqing, China	Yes	Evaluated 278 pregnant women and 79 infants for CT. Vertical transmission of CT was 55% (11/22). Neonatal conjunctivitis (45% versus 18.3%) and pneumonia (30% versus 8.3%) were more common in infants of CT-infected women compared to CT-uninfected women (*p* < 0.05). DNA sequencing of CT isolated from mothers and infants was identical
				
Yu et al., 2009 [30]	Asia	Chongqing, China	Yes	300 pregnant women were evaluated for CT and 11% found to have CT. Vertical transmission of CT was 24% and was higher for vaginal delivery 66.7% versus cesarean 8.3%
				
Chotnopparatpattara et al., 2003 [49]	Asia	Bangkok, Thailand	No	Evaluated 182 pregnant women at >37 weeks' gestation and followed up their infants for 2 months after delivery. None of the newborn infants had CT, and none had CT at the 2-month follow-up
				
Laga et al., 1986 [13]	Africa	Nairobi, Kenya	Yes	Evaluated infants for CT and NG ophthalmia neonatorum and found rates of 23.2 per 100 live births of ON; 8.1 per 100 live births or 31% of 181 cases of neonatal conjunctivitis were from CT. For 201 CT-exposed infants, CT was recovered from the eye in 31% and throat in 2% of infants. NG transmission to infants was more likely in infants whose mothers had both CT and NG
				
Datta et al., 1988 [61]	Africa	Nairobi, Kenya	Yes	Evaluated CT-exposed and CT-unexposed infants for CT, ophthalmia neonatorum or conjunctivitis, and pneumonia. CT-exposed infants were more likely to have CT positive cultures (37% (18/49) versus 0% (0/40)), develop ophthalmia neonatorum or conjunctivitis (37% versus 15%, *p* = 0.04), or have pneumonia (12% versus 0%, *p* = 0.05). One CT-exposed infant death was also noted

*CT and conjunctivitis/neonatal ophthalmia* (studies = 11)				

Khauv et al., 2014 [62]	Asia	Siem Reap, Cambodia	Yes	Evaluated 54 cases of acute eye infections of children (6 DOL to 16 yo) presenting to an ophthalmology clinic. Of the 10 cases of ophthalmia neonatorum, 60% were from CT
				
Wu et al., 2003 [63]	Asia	Chongqing, China	Yes	Evaluated 125 infants with neonatal conjunctivitis for CT and recovered CT in 41.6% of infants by cell culture and in 51.2% of infants by PCR
				
Yip et al., 2007 [64]	Asia	Hong Kong, China	Yes	Evaluated 192 infants with neonatal conjunctivitis for CT. CT was isolated from 12.5% (24) of cases and NP colonization with CT was also found in 62.5% (15) of these cases. CT conjunctivitis incidence was estimated at 4 per 1000 live births. Only one infant had treatment failure after being treated with oral erythromycin
				
Jhon and Chang, 1989 [65]	Asia	Central Taiwan, China	Yes	Evaluated 98 newborn infants with conjunctival secretions along with 122 children with respiratory tract disease in the hospital for CT. CT was recovered from 12.2% (12) of infants' eyes and 26.2% (32) of respiratory secretions
				
Ng et al., 1987 [66]	Asia	Singapore	No	Cases of ophthalmia neonatorum were retrospectively reviewed and only 2 cases from CT were found
				
Sergiwa et al., 1993 [67]	Asia	Bangkok, Thailand	Yes	17 cases of neonatal conjunctivitis were evaluated for an etiology. CT was recovered in 29.4% (5) of cases and a statistically significant association with CT was noted
				
Buisman et al., 1988 [68]	Africa	Ndoungué, Cameroon	Yes	449 newborn infants were examined for 1 mo to evaluate for ophthalmia neonatorum. Ophthalmia neonatorum (ON) occurred in 19.4% of cases with CT ON diagnosed in 1.8% (8) of infants, which were more severe
				
Frost et al., 1987 [69]	Africa	Franceville, SE Gabon	Yes	Evaluated infants for ophthalmia neonatorum over a 7 mo period, and CT was isolated from 2.7% (17) infants. Conjunctivitis from CT was usually unilateral as opposed to bilateral
				
Mabey et al., 1987 [70]	Africa	Gambia	Yes	112 infants with ophthalmia neonatorum were evaluated for CT and NG; CT was detected in 33% (37). Also followed 335 neonates and found 16% (55) developed ophthalmia neonatorum with 16% of cases due to CT
				
Datta et al., 1994 [71]	Africa	Meru, Central Kenya	Yes	Evaluated 38 infants with ophthalmia neonatorum and 277 children with trachoma in a trachoma endemic region. CT was isolated in 8-9% of infants with ophthalmia neonatorum, and chlamydia was isolated from 31% of children with trachoma with 92% belonging to classic trachoma serovars. The study did not support that perinatal CT ophthalmic infections played a major role in trachoma epidemiology
				
Fransen et al., 1986 [72]	Africa	Nairobi, Kenya	Yes	149 infants with ophthalmia neonatorum were evaluated for CT, NG, and other infections. CT was isolated from 13% of infants, and 3/5 were trachoma serovars

Donders et al., 1991 [59], Paul et al., 1999 [38], and Christian et al., 2005 [43]—see Table 1 on adverse pregnancy outcomes for other information relevant to conjunctivitis/neonatal ophthalmia. Laga et al., 1986 [13]—see vertical transmission section above in this table.				

*CT and pneumonia* (studies = 8)				

Ngeow et al., 1997 [73]	Asia	Kuala Lumpur, Malaysia	No	87 children (ages 2 mo to 3 yrs) admitted to a hospital were evaluated for the etiology of their lower respiratory tract infection (LRTI). CT was uncommon and only recovered in 1.2% of patients and in only 1 patient under 6 mo (5.9%)
				
Puthavathana et al., 1994 [74]	Asia	Bangkok, Thailand	Yes	76 infants < 6 mo of age presenting to hospitals were evaluated for CT and viruses. CT was isolated in 16.7% of infants in one hospital and 21.7% of infants in another; overall CT was recovered in 18.5% of all infants with LRTI from both sites
				
Ekalaksananan et al., 2001 [75]	Asia	Khon Kaen, Thailand	Yes	74 children < 5 yrs admitted to the hospital for LRTI were evaluated for infectious etiologies. For infants < 1 yr, 10% had CT recovered from nasopharyngeal aspirates and were diagnosed with CT pneumonia
				
Pientong et al., 2011 [76]	Asia	Khon Kaen, Thailand	Yes	170 children (1 mo to 2 yrs) admitted to the hospital for acute bronchiolitis were evaluated for CT, respiratory viruses, and other etiologies. 2.4% had CT, and all of these children also had RSV
				
Muhe et al., 1999 [77]	Africa	Addis Ababa, Ethiopia	Yes	Evaluated 405 infants < 3 mo of age presenting to a hospital as part of multicenter WHO study on pneumonia, sepsis, and meningitis. In 203 infants that had nasopharyngeal aspirates done, 15.8% had CT isolated whereas 28% had RSV isolated
				
Forgie et al., 1991 [78]	Africa	Gambia	No	90 infants < 1 yo with pneumonia and 43 controls were evaluated for viral and other infectious etiologies. CT was also isolated from 2 infants with lower respiratory tract infection and one control patient, whereas RSV was found in 37% of patients
				
Were et al., 2002 [79]	Africa	Nairobi, Kenya	Yes	Evaluated 52 infants between 7 and 30 days of life to determine prevalence of CT-associated pneumonia and found 63.5% (33/52) had CT isolated from their upper airways and 51% (24/47) had CT-associated pneumonia based on findings of both CT-colonization and interstitial pneumonia on X-rays
				
Zar 2005 [80]	Africa	Cape Town, South Africa	Yes	Evaluated 100 ambulatory infants with signs of lower respiratory tract infection and found 6% had CT infection. Infants with CT were younger (mean age: 3.8 weeks versus 8.7 weeks, *p* = 0.03) and were more likely to have eye discharge (*p* = 0.02) or conjunctivitis (*p* = 0.01) than uninfected infants

Datta et al., 1988 [61], Shen et al., 1995 [33], and Wu et al., 1999 [34], also discuss rates of pneumonia in CT-exposed infants in the CT vertical transmission section of this table.				

*Other adverse infant outcomes* (studies = 3)				

Chaisilwattana et al., 1997 [50]	Asia	Bangkok, Thailand	No	Secondary analysis of a multicenter perinatal HIV transmission study testing pregnant women midpregnancy for CT and NG. HIV MTCT was similar for women with and without CT (24.1% versus 23.2%, *p* = 0.9)
				
Adachi et al., [60]	Africa	South Africa^*∗*^	Yes	A secondary analysis of a large, randomized, multicenter trial of HIV-infected pregnant women evaluating different antiretroviral prophylaxis regimens to prevent intrapartum HIV MTCT. Infants of women coinfected with CT and NG had higher rates of adverse outcomes (sepsis, pneumonia, congenital syphilis, septic arthritis, conjunctivitis, LBW, preterm birth, or death) compared to infants of women uninfected with these STIs (65.7% versus 37%, *p* = 0.001) and were especially at risk for death, LBW, and preterm delivery. Death (11.4% versus 3%, *p* = 0.02), low birth weight (42.9% versus 16.9%, *p* = 0.001), and preterm delivery (28.6% versus 10.2%, *p* = 0.008) were higher among infants of CT and/or NG coinfected women compared to STI unexposed infants. These infants born to mothers with CT and/or NG were 1.4 times more likely to have at least one of these adverse outcomes (OR 1.35, 95% CI 1.03–1.8)
				
Adachi et al., 2015 [24]	Africa	South Africa^*∗*^	Yes	Additional secondary analysis of the RCT noted above. Among the 117 cases of HIV MTCT, higher rates of HIV MTCT were noted among women with CT (10.7%) or with both CT and NG (14.3%) compared to those uninfected 8.1% (*p* = 0.04); findings suggested a possible association of CT with increased HIV MTCT (OR 1.47, 95% CI 0.9–2.3, and *p* = 0.09)

^*∗*^Adachi et al. [24, 60]—30% of cohort from South Africa.

STI = sexually transmitted infection, CT = *Chlamydia trachomatis*, NG = *Neisseria gonorrhoeae*, and RSV = respiratory syncytial virus.

yo = year(s) old, yrs = years, mo = month(s), DOL = days of life, MTCT = mother-to-child transmission, and LBW = low birth weight.

LRTI = lower respiratory tract infection, RCT = randomized clinical trial, NP = nasopharyngeal, and ON = ophthalmia neonatorum.

**Table 3:** Studies from sub-Saharan Africa and Asia on *Chlamydia trachomatis *screening and treatment in pregnancy to prevent adverse pregnancy and infant outcomes.

CT screening/treatment to prevent adverse pregnancy and infant outcomes (total studies = 2)				

*CT screening/treatment to prevent adverse pregnancy outcomes* (studies = 2)				

Study	Region	Country	Support benefit	Findings

Gray et al., 2001 [26]	Africa	Rakai, Uganda	Yes	Evaluated 2070 pregnant women in an analysis from a cluster-randomized STI presumptive treatment trial with azithromycin, cefixime, and metronidazole (also included benzathine penicillin G if syphilis serology was positive) versus placebo. Found reduction in CT/NG (RR 0.43, 95% CI 0.27–0.68) and other STIs. Found reduction in low birth weight (RR 0.68, 95% CI 0.53–0.86), preterm delivery (RR 0.77, 95% CI 0.56–1.05), neonatal death, (RR 0.83, 95% CI 0.71–0.97), and infant ophthalmia (RR 0.37, 95% CI 0.20–0.70).
				
Rastogi et al., 2003 [39]	Asia	New Delhi, India	Yes	Erythromycin was given to 17 CT-infected pregnant women and compared to 42 untreated CT-infected women lost to follow-up and 269 women without CT. CT-infected and treated women had infants with higher mean gestational ages at the time of delivery (35.5 versus 33.1 weeks, *p* < 0.05) and higher birth weights (2200 versus 2113.3 g, although not significant) in comparison to untreated women. Stillbirths were higher in CT-infected and untreated women in comparison to the CT-uninfected (11.5% versus 4.7%), and 0% in CT-infected treated women.

*CT screening/treatment to prevent adverse infant outcomes* (studies = 0)				

None^*∗*^				

^*∗*^Study by Gray et al. [26] includes some adverse infant outcomes—neonatal death and infant ophthalmia as noted above.

CT = *Chlamydia trachomatis*, NG =* Neisseria gonorrhoeae*, STI = sexually transmitted infection.
